# Examining the Aryl Hydrocarbon Receptor Network in the Placental Tissues of Pregnancies Complicated by Pre-Eclampsia: An Explorative Case–Control Analysis

**DOI:** 10.3390/life13112122

**Published:** 2023-10-26

**Authors:** Serena Xodo, Ambrogio P. Londero, Maria Orsaria, Stefania Marzinotto, Gianluca Colussi, Angelo Cagnacci, Laura Mariuzzi, Giorgia Gri

**Affiliations:** 1 Clinic of Obstetrics and Gynecology, “Azienda Sanitaria Universitaria Integrata di Udine”, DAME, University of Udine, 33100 Udine, Italy; 2 Department of Neuroscience, Rehabilitation, Ophthalmology, Genetics, Maternal and Infant Health, University of Genoa, 16132 Genova, Italy; angelo.cagnacci@unige.it; 3 Obstetrics and Gynecology Unit, IRCCS Istituto Giannina Gaslini, 16147 Genova, Italy; 4 Institute of Pathology, “Azienda Sanitaria Universitaria Integrata di Udine”, DAME, University of Udine, 33100 Udine, Italy; mariaorsaria@yahoo.it (M.O.);; 5 Hypertension Unit, Department of Medicine, ASFO “Santa Maria degli Angeli” Hospital of Pordenone, 33170 Pordenone, Italy; gianluca.colussi@asfo.sanita.fvg.it; 6 Academic Unit of Obstetrics and Gynecology, IRCCS Ospedale San Martino, 16132 Genoa, Italy; 7 DIMEC—Department of Medical and Surgical Sciences, University of Bologna, 40126 Bologna, Italy; giorgia.gri@unibo.it

**Keywords:** pre-eclampsia, aryl hydrocarbon receptor, AhR, IDO1, S100A4, CYP1B1, TiPARP

## Abstract

Severe maternal and newborn morbidity and mortality associated with pre-eclampsia, which are caused partly by premature delivery, affect a factual proportion of pregnancies. Despite its prevalence, the underlying causes of pre-eclampsia remain elusive, with emerging evidence implicating the aryl hydrocarbon receptor (AhR) in its pathogenesis. This study sought to elucidate the involvement of the AhR and its associated pathway in pre-eclampsia by comparing placental components of the AhR pathway in pregnant individuals with and without pre-eclampsia. This case–control investigation was conducted at the University Hospital of Udine from May 2021 to February 2023. The AhR was assessed using immunohistochemistry and immunofluorescence, and its mRNA was evaluated using a Real-Time Quantitative Reverse Transcription PCR. Levels of mRNA expression were also estimated for other components of the AhR pathway (CYP1B1, IDO1, ARNT, TIPARP, S100A4, and AHRR). Our findings show decreased levels of expression of AhR, IDO1, ARNT, TiPARP, and S100A4 in the placental tissues of individuals with pre-eclampsia compared to controls (*p* < 0.05). The AhR exhibited a distinct localization within the syncytiotrophoblast (nuclei and cytoplasm) and CD45-positive cells (nuclei and cytoplasm). Furthermore, a significant positive correlation between the AhR and S100A4 (rho = 0.81) was observed in normal placentas, while CYP1B1 displayed a significant negative correlation with the AhR (rho = −0.72), within addition to its negative correlation with TiPARP (rho = −0.83). This study illuminates pre-eclampsia’s molecular aberrations, suggesting new diagnostic, therapeutic, and mechanistic approaches. This study emphasizes the need for more research to validate and broaden these findings to improve the management of this complex pregnancy condition.

## 1. Introduction

Pre-eclampsia appears to have a significant influence on preterm birth, in addition to other causes [1,2]. Davies et al. found that pre-eclampsia (PE) significantly contributes to preterm birth, especially iatrogenic preterm birth [2]. Moreover, they argued that the successful management of PE could substantially reduce the incidence of premature birth [2]. It is estimated that PE complicates up to 8% of all pregnancies [3]. There is no single theory on the etiopathogenesis of PE, and the therapeutic approaches proposed reflect the diversity of hypotheses [4,5,6,7,8,9,10,11]. Even though there is no agreement among experts on the mechanisms underlying the pathogenesis of PE, most recognize that this syndrome has two different phenotypes [4,5,6,7,8,9,10,11]. Early-onset PE is generally attributed to shallow endovascular trophoblast invasion in the spiral arteries with secondary poor placentation, which might explain the presence of fetal growth restriction in some cases. In contrast, late-onset PE, which generally appears after 34 weeks of gestation, is strongly associated with maternal constitutional and lifestyle-related factors, thus representing the majority of PE cases in developed countries [12]. It is now clear that the different pathologic processes activate a common pathway consisting of endothelial cell activation, intravascular inflammation, and syncytiotrophoblast stress [13].

Recently, immune maladaptation has gained increasing importance as a putative etiologic factor involved in the pathogenesis of early PE. Interestingly, some authors have theorized that from an evolutionary perspective, the deep trophoblastic invasion characteristic of human placentation could have much to do with the increasing amount of blood required during gestation by the human fetus, especially by its brain, which is of a larger size compared to other animal species. An immunological compromise is needed between the maternal immune system and the semi-allogenic trophoblast at the feto-maternal interface to achieve normal hemochorial placentation. Regulatory T cells (Tregs) are a specialized population of T cells that induce tolerance to the semi-allograft fetus. T helper (Th) cells cooperate in modulating systemic immune responses. Recent studies have shown that contrary to what occurs in a healthy pregnancy, in PE, the balance between these cell populations shifts toward a predominance of Th1 cells (which are essential in cell-mediated immunity: autoimmune diseases can be the result of an overly robust immune response) and Th17 cells (which are essential for immune responses at mucosal surfaces; excessive activity has been linked to chronic inflammation and autoimmune disease) cells and a decrease in Treg cells (which are responsible for suppressing excessive immune responses and essential for sustaining immune tolerance) [14,15].

**Figure 1:**
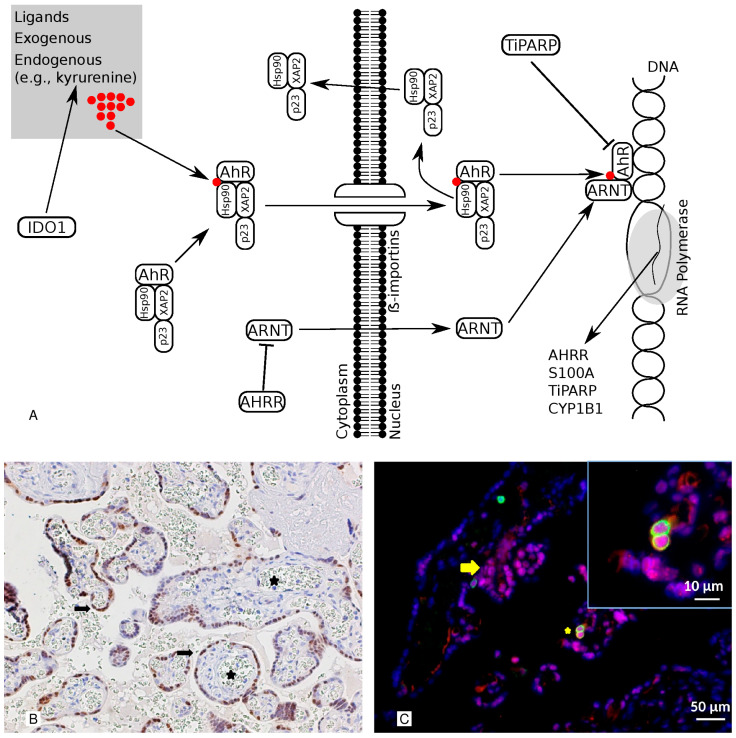
This illustration shows the pathway and the cellular localization of the AhR. Panel (**A**) The AhR pathway is illustrated. IDO1 is an enzyme that can stimulate the production of kynurenine, one of the AhR’s endogenous ligands. The ARNT is the nuclear companion of the AhR, whereas the AHRR is the AhR’s endogenous repressor. TiPARP has a modulatory influence on the AhR via the TIPARP-dependent repression ADP-ribosylation of AhR. CYP1B1 and S100A are target genes. Panel (**B**) Representative images of the immunohistochemical staining of AhR in healthy, term placentas. The AhR is detected in the nuclei of the cell layer lining the villi (syncytiotrophoblast) (arrows) but not in the fetal capillaries (stars). 200× magnification. Panel (**C**) A double-marker immunofluorescence analysis of the AhR and CD45 in sections from the placenta. The AhR is detected in the nuclei and cytoplasm of the syncytiotrophoblast (red; arrow) and CD45-positive cells (green; star). In the inset is a detail at higher magnification. Moreover, the red-labeled AhR nuclear localization results in a purple signal due to its overlap with the blue DAPI staining. Indeed, we observed a significant amount of AhR protein expressed in the nuclei of syncytiotrophoblasts, as revealed via Pearson’s colocalization coefficient for the cellular distribution of the AhR, which equals 0.71. Bars 50 μm and 10 μm (inset).

The aryl hydrocarbon receptor (AhR) is a transcription factor that many structurally diverse ligands can activate, and it is a central player in T cell differentiation. It is additionally involved in the regulation of the function of placental endothelial and trophoblast cells [16]. Several genes are known to be involved in the AhR pathway (Figure 1A) [17,18,19]. Indoleamine 2, 3-dioxygenase 1 (IDO1) is an enzyme that can stimulate the production of kynurenine, one of the AhR’s endogenous ligands. Aryl Hydrocarbon Receptor Nuclear Translocator (ARNT) is the AhR’s nuclear partner, whereas the Aryl Hydrocarbon Receptor Repressor (AHRR) functions as the AhR’s endogenous repressor. TCDD-Inducible Poly(ADP-Ribose) Polymerase (TiPARP) modulates the AhR via the ADP-ribosylation-dependent repression of the AhR. S100 calcium-binding protein A4 (S100A) and Cytochrome P450 1B1 (CYP1B1) are target genes. Although previous reports implicated this pathway in the mechanisms of PE [20,21], it is still unknown to what extent the AhR is involved. In order to determine the role of the AhR and its pathway in PE, this study compared the placental AhR pathway in pregnant women with and without PE at birth.

## 2. Materials and Methods

### 2.1. Study Design, Setting, and Sample

This case–control investigation was carried out at the University Hospital of Udine. Samples were gathered between May 2021 and February 2023. The regional review board and the hospital’s clinical research center approved the current study (protocol number: asufc/2021/0044374), which complied with the requirements of the Italian Data Protection Authority’s general authorization to process data for scientific research purposes. Moreover, all ethical principles of the Helsinki Declaration [22] were followed. The inclusion criteria for the cases were PE-diagnosed singleton pregnancies. Unaffected, uncomplicated pregnancies were randomly selected as controls. Exclusion criteria included concurrent pre-gestational or gestational maternal or fetal pathologies, pregnancies complicated by viral or bacterial infection, smoking during pregnancy, and known fetal chromosomal abnormalities. Twenty singleton pregnancies, ten with PE and ten controls, were analyzed.

### 2.2. Data and Tissue Collection

After informing the women and obtaining their written informed consent to participate, recruitment and data collection occurred. In this study, residents and obstetricians collected information about maternal and pregnancy characteristics. The following clinical data were collected: maternal age, parity, tobacco smoke, pre-pregnancy body mass index (BMI), medical history, pregnancy complications, mode of delivery, and neonatal health.

The central and paracentral regions of the placenta were sampled in two distinct areas, specifically targeting the villi, as previously documented [23]. The samples were prepared according to the following protocol for an RNA analysis. The handling of the samples for immunohistochemistry and immunofluorescence analyses was conducted in accordance with the following procedures.

Neonatal weights were classified as small for gestational age below the 10th centile (SGA, birthweight 10th centile) and small for gestational age below the third centile (SGA, birthweight < 3rd centile) based on Italian growth post-natal standards [24]. As previously described [23,25], neonatal weight was also evaluated as a multiple of the median (MoM). The neonatal weight MoM is the ratio between the observed birthweight and the 50th percentile of birthweight at the same gestational age (specific to gender and parity) [24,25]. As previously defined, the placental index was calculated by dividing placental weight in grams by birth weight in grams [26]. Hypertension was defined as diastolic blood pressure ≥ 90 mmHg or systolic blood pressure ≥ 140 mmHg [26,27]. Pre-eclampsia was defined as the presence of hypertension (as defined above) accompanied by proteinuria or end-organ dysfunction after 20 weeks of gestation [26,27]. Proteinuria was defined as previously indicated (urinary protein of more than 0.3 g in 24 h) [26,27].

### 2.3. Histopathology, Immunohistochemistry, and Immunofluorescence

For a histopathological analysis and immunohistochemistry, formalin-fixed, paraffin-embedded tissues were processed as 4 μm thick transverse sections, as previously described [23]. After that, the slides were stained with H&E. Moreover, immunohistochemistry sections were also prepared. For antigen retrieval, the specimens were heated in Target Retrieval Solution (at a low pH, K8005; DAKO, Glostrup, Denmark) with PT-link (DAKO) at 98 °C for 20 min. The slides were then incubated for 10 min at room temperature in hydrogen peroxide to inhibit endogenous peroxidase activity. Before administering the primary antibody, phosphate-buffered saline (PBS) was used to rinse the tissue. The sections were then incubated with anti-AhR (LS-A3391, LS-Bio, Rabbit polyclonal) in a moist chamber at room temperature for one hour. EnVision (Rabbit/Mouse K5007; Dako) was used as the secondary antibody, and 3,3′-diaminobenzidine + chromogen (K5007; Dako) was used to detect positive staining. The sections were counterstained with hematoxylin and then coverslipped. We used the slides incubated with nonimmune rabbit serum instead of the primary antibody as the negative control. As positive controls, the manufacturer-suggested controls were utilized. M.O. and L.M., two independent pathologists, evaluated each histopathological and immunohistochemical staining. As previously described, a semiquantitaive immunohistochemical staining analysis was performed using the H-score for the nuclei and the intensity score for the cytoplasm of the syncytiotrophoblast [10]. In the event of a disagreement, both pathologists examined the specimen and agreed on it.

For an in situ double-marker immunofluorescence analysis of the placenta, two sequential cycles of single-marker immunostaining were performed as previously reported [18,19]. To determine the presence of AhR within the immune cells in addition to the syncytiotrophoblast or cytotrophoblast, fluorescent anti-AhR and anti-CD45 antibodies were used to stain fixed placental sections. CD45 is a transmembrane protein found on all differentiated hematopoietic cells except for erythrocytes and plasma cells [28]. Hofbauer cells, or fetal macrophages, also express CD45 in the placenta [28]. Anti-AhR (LS-A3391, LS-Bio, polyclonal rabbit) and anti-CD45 (Dako, Clone 2B11, monoclonal mouse) were utilized. Following this, A555 and A488 donkey–rabbit fluor-conjugated secondary antibodies (Molec. Probes, Invitrogen) were employed. The slides were examined using a Leica DMI 6000B coupled to a CCD camera (Leica DFC350FX). Two expert pathologists (M.O. and L.M.) blindly evaluated the tissue sections. In the event of a dispute, both pathologists examined the specimen and agreed upon a conclusion. Nuclear co-localization was assessed as previously described, using ImageJ software (v1.53k, http://imagej.nih.gov/ij) [29].

### 2.4. Real-Time Quantitative Reverse Transcription PCR (qRT-PCR)

All samples were transferred to 500 μL of TRIzol Reagent (Invitrogen) and processed according to the manufacturer’s instructions for total RNA isolation as previously decribed [30]. RNA (1 g) was reverse-transcribed using a SuperScript^®^ III REV (Life Technologies, Carlsbad, CA, USA) transcript. Per the manufacturer’s instructions, the quantitative RT-PCR reaction was conducted using a Roche LightCycler^®^ 480 and an SSOADV UNIVER SYBR GRN SMX 500 (BIO-RAD, Hercules, CA, USA). The AhR, Cytochrome P450 1B1 (CYP1B1), Indoleamine 2, 3-dioxygenase 1 (IDO1), Aryl Hydrocarbon Receptor Nuclear Translocator (ARNT), 2,3,7,8- tetrachlorodibenzo-p-dioxin (TCDD) Inducible Poly(ADP-Ribose) Polymerase (TiPARP), S100 calcium-binding protein A4 (S100A4), and Aryl Hydrocarbon Receptor Repressor (AHRR) genes were included because they correlate with the function of the AhR. The glyceraldehyde-3-phosphate dehydrogenase (GADPH) gene was used as a normalizer in each sample. The following primer sequences were designed using the National Center for Biotechnology Information (NCBI) Primer Blast: GADPH forward: 5#-GTCTCCTCTGACTTCAACAGCG-3#; GADPH reverse: 5#-ACCACCCTGTTGCTGTAGCCAA-3#; AhR forward: 5#-ACATCACCTACGCCAGTCGC-3#; AhR reverse: 5#TCTATGCCGCTTGGAAGGAT-3#; CYP1B1 forward: 5#-TATCCTGATGTGCAGACTCG-3#; CYP1B1 reverse: 5#TCCTTGTTGATGAGGCCATC-3#; IDO1 forward: 5#-TTCAGTGCTTTGACGTCCTG-3#; IDO1 reverse: 5#TGGAGGAACTGAGCAGCAT-3#; ARNT forward: 5#-GCTGCTGCCTACCCTAGTCTCA-3#; ARNT reverse: 5#TCTGCTGTCCGTGTCTGGAA-3#; TIPARP forward: 5#-GGCAGATTTGAATGCCATGA-3#; TIPARP reverse: 5#TGGACAGCCTTCGTAGTTGGT-3#; S100A4 forward: 5#-GTGCAGTTCTCTGGAGCAGG-3#; S100A4 reverse: 5#GCTTGAACTTGTCGCCCTCT-3#; AHRR forward: 5#-CACCAGTCTGTGCGAATCGGAA-3#; and AHRR reverse: 5#CAGTCTGTTCCCTGAGCACCAA-3#. The reactions were carried out in triplicate in three separate experiments. The quantification of mRNA was expressed as the cycle threshold (Ct). The means of the Ct values from each triplicate sample were calculated and used for further analyses. ΔCt(gene) = Ct(gene) − Ct(GAPDH) values were used to represent the differences between the Ct values of the tested genes and those of the reference gene.

### 2.5. Data Analysis

In this investigation, R v4.3.0 (R Foundation for Statistical Computing, Vienna, 2023) was used to carry out the data analysis [31]. A *p*-value of less than 0.05 (*p* < 0.05) was considered statistically significant. The RT-PCR data were presented as ΔCT expression values (i.e., 2^(−ΔCT)^). The Kolmogorov–Smirnov test was used to evaluate the distribution of continuous variables for normality. This text provides continuous values for parametric distributions as means (±standard deviations) and for non-parametric distributions as medians and interquartile ranges (IQRs). Percentages and absolute values were used to represent category values. The *t*-test (Student’s t) was used to evaluate differences between groups for variables with a normal distribution; meanwhile, the Wilcoxon test was used for variables with a non-parametric distribution. Additionally, Spearman’s test was utilized to assess the correlations between the variables under consideration. Positive and negative Spearman correlation coefficients were considered weak between 0.10 and 0.30, moderate between >0.30 and 0.60, strong between >0.60 and 0.90, and ideal above 0.90 [32]. For categorical variables, differences between groups were determined using the chi-squared or Fisher’s exact test as appropriate.

## 3. Results

### 3.1. Population Description

The median maternal age was 33.50 years (IQR 32.25–36.25) in the PE group, which was not significantly different from the control group (31.00 IQR 29.25–32.75). Additional population characteristics are illustrated in Table 1. Half of the women were nulliparous in both groups. The gestational age at childbirth significantly differed among the groups, with an increased prevalence of preterm delivery in the PE patients (33.50, IQR 31.25–35.75 vs. 38.50, IQR 38.00–39.75). Most of the women in the control group delivered vaginally (8/10), while the prevalent delivery mode in the PE patients was cesarean section (7/10). Regarding fetal growth, no SGA babies were retrieved in the control group, whereas 2/10 SGA < 3rd percentile and 3/10 SGA < 10th percentile were observed in the PE group. This difference is better reflected by the mean neonatal weights of the two groups, 1746.50 g (IQR 1166.00–2643.75) vs. 3043.00 g (IQR 2849.00–3341.00), but not by the difference in the neonatal weight MoM between the groups (0.87, IQR 0.73–0.96 vs. 0.93, IQR 0.89–0.98). Placental weight was significantly decreased in the PE patients compared with the controls (265.00 g, IQR 251.25–505.75 vs. 597.50, IQR 502.50–678.25), but the placental index was not (0.21, IQR 0.18–0.25 vs. 0.19, IQR 0.17–0.19). The Apgar scores at the first and fifth minutes were 7 and 9 in the PE group and 8.5 and 9 in the control group.

### 3.2. AhR Localization

Using immunohistochemistry (IHC) and immunofluorescence (IF), samples of term placentas were examined to identify the location and cells expressing the AhR. Positive staining for the AhR molecule was predominantly observed in the syncytiotrophoblast nuclei (at the cell layer bordering the villi) (Figure 1B) but not in the fetal capillaries. In immunofluorescence, the expression pattern of the AhR resembles the pattern observed via immunohistochemistry. Notably, the localization was not only nuclear but also cytoplasmic and granular, indicating that not all of the AhR is in an active form. Figure 1C demonstrates that CD45-positive immune cells were also highly positive for the AhR, which was expressed in both the cytoplasm and nucleus.

### 3.3. qRT-PCR Data

Differences in gene expression patterns between the two groups are presented in Table 2 as delta CT expression values. Of note, the AhR, IDO1, the ARNT, TiPARP, and S100A4 turned out to have significantly reduced levels of expression in the PE patients compared with the normal controls. By contrast, CYP1B1 and the AHRR were only slightly increased in the PE patients compared to the controls.

### 3.4. AhR Immunohistochemical Expression

A semiquantitative analysis of the IHC expression of the AhR showed a reduced level of expression in pre-eclampsia cases. The nuclear H-score in pre-eclampsia syncytiotrophoblast was significantly lower than in the controls (8.50, IQR 0.50–15.00 vs. 154.00, IQR 145.00–225.25, *p* < 0.05). Moreover, the intensity score of the syncytiotrophoblast in pre-eclampsia was significantly lower than the in controls (0.50, IQR 0.00–1.00 vs. 1.00, IQR 1.00–1.75, *p* < 0.05).

### 3.5. Correlations

**Figure 2:**
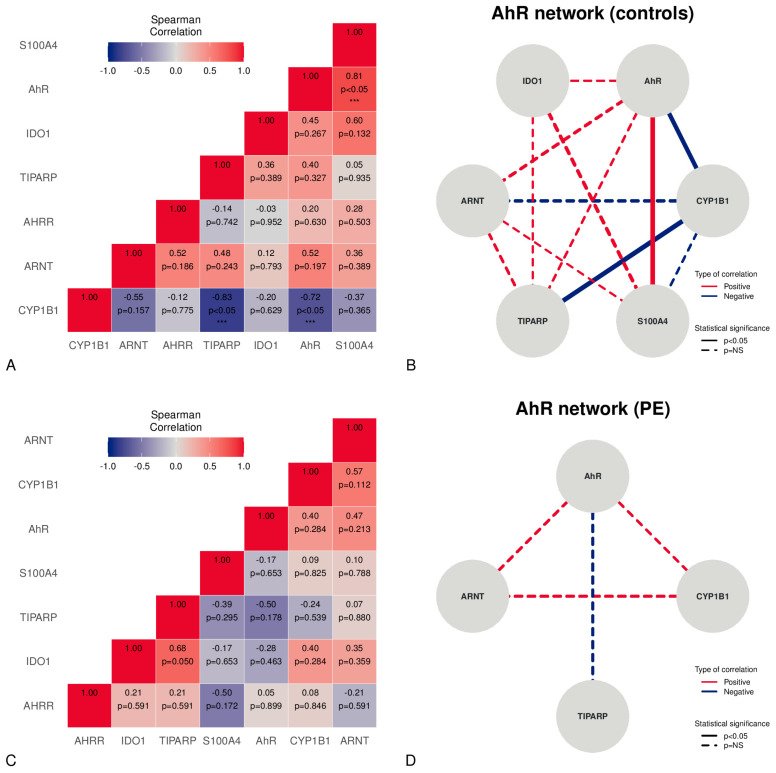
Correlations between mRNA expression levels in the placentas studied, stratified v controls and PE. The correlation matrix in the controls is shown in Panel (**A**). The network figure in Panel (**B**) depicts the AhR-correlated mRNA expression network in controls. Panel (**C**) represents the PE correlation matrix. The network figure in Panel (**D**) illustrates the AhR-correlated mRNA expressions network in PE. *** = statistically significant (*p* < 0.05).

A correlation analysis applied to placental samples from normal pregnancies revealed the presence of significant strong, positive correlations between the AhR and S100A4 (Figure 2A,B). Moreover, a significant strong, negative correlation was found between CYP1B1 and the AhR or TiPARP (Figure 2A,B). Although PE showed no significant correlations, a moderate positive, non-significant relationship, according to the Spearman coefficient, was found between the AhR, ARNT, and CYP1B1 (Figure 2C,D). Moreover, a moderate negative, non-significant relationship between the AhR and TiPARP was observed (rho −0.50) (Figure 2C,D).

## 4. Discussion

### 4.1. Main Results

This study showed that pre-eclamptic women had decreased levels of expression of the AhR, IDO1, the ARNT, TiPARP, and S100A4 in their placental tissues compared with controls. The AhR was localized in the syncytiotrophoblast (in the nuclei and cytoplasm) and CD45-positive cells (in the nuclei and cytoplasm). Moreover, in normal placentas, the AhR was significantly positively correlated with S100A4, and CYP1B1 was significantly negatively correlated with the AhR and TiPARP. The semiquantitative immunohistochemical expression of the AhR was also significantly reduced in pre-eclamptic placentas.

### 4.2. Results in the Context of What Is Known

Few studies have found high levels of expression of the AhR and ARNT in term placentas, mainly in trophoblast syncytium cells and the endothelial cells of veins and umbilical arteries [33,34]. We found that the AhR is expressed in the syncytiotrophoblast but not in the endothelial cells. In addition, we demonstrated strong staining for the AhR in placental immune cells that was not shown previously.

The aryl hydrocarbon receptor is a transcription factor expressed in multiple tissues which is known to respond to environmental toxins, such as TCDD (2,3,7,8-Tetrachlorodibenzo-p-dioxin), as well as to endogenous ligands, including heme and tryptophan metabolites [19]. Upon binding to its ligands, the AhR–ligand complex translocates into the nucleus, where the complex binds to the ARNT to form a heterodimer which activates the dioxin-response element, inducing the expression of many downstream genes (e.g., CYP1B1 and TiPARP) (Figure 1A). Several negative feedback loops tightly regulate the AhR signaling pathway. The AhR, for instance, can be transported to the cytosol, where it is degraded via the 26S proteasome pathway [35]. AhR ligands can be metabolized by CYP1B1 [17]. Alternatively, it can be inhibited by an AhR repressor (AHRR), which binds competitively to the ARNT and reduces AhR activity [17,35]. Additionally, TiPARP has a modulatory effect on the AhR via the ADP-ribosylation-dependent repression of the AhR [17]. It is interesting to note that a significantly higher level of expression of the AhR was found in normal placentas than PE. Furthermore, a significant correlation between the AhR and the transcription target gene S100A4 and a negative correlation between the AhR and CYP1B1, which is a target gene and a negative feedback regulator, were reported. Our findings suggest that the AhR pathway and its regulator feedback are functional in a healthy placenta. The previous literature hypothesized a role for AhR pathway activation/up-regulation in the protective effect of smoking against PE [20,21]. In accordance with this hypothesis, we found a low level of expression of the AhR pathway in PE patients and a high level of expression in the controls; therefore, the modulation of the AhR pathway can explain the protective effect of cigarette smoking against PE.

The AHR is best known for its ability to mediate the toxic responses of the environmental contaminant TCDD. Among the target genes of the AhR is cytochrome P450 1B1, which exhibits multifunctional activities in diverse metabolic pathways. The role of CYP1B1 has been extensively investigated in glaucoma and cancers, and it was recently found that this protein is actively involved in the development of hypertension through endothelial dysfunction related to ROS [36,37]. Although its negative correlation with the AhR in normal placentas is probably due to its role as negative feedback, it remains unclear how the downregulation of this protein is linked to PE, as demonstrated by our data.

Another target gene of the AhR is TCDD-inducible poly(ADP-ribose) polymerase (TiPARP). ADP-ribosylation is a ubiquitous post-translational protein modification which is crucial for various cellular processes, including DNA repair, transcription, apoptosis, cell proliferation, and cell death. TiPARP inhibits the transactivation of the AhR by promoting its proteolytic degradation via ribosylation, suggesting that it is implicated in the AhR’s negative feedback [38]. Our data indicate that TiPARP is substantially downregulated in preeclamptic women, which is likely related to the decreased expression of the AhR pathway in this condition. Its negative correlation with CYP1B1 in the normal placenta suggests the presence of complex mechanisms for negative feedback regulation within this pathway.

IDO1 is involved in kynurenine production, and kynurenine is an endogenous ligand of the AhR, leading to its activation. Kynurenine derives from the catabolism of the essential amino acid tryptophan. The degradation of tryptophan occurs predominantly along the kynurenine pathway in which the rate-limiting step is the conversion of tryptophan into kynurenine which is regulated by hepatic tryptophan 2,3-dioxygenase (TDO) or IDO1. Generally, IDO1 expression increases in response to inflammatory stimuli but in pregnancy, the most abundant source of IDO1 is the placenta, specifically its endothelial cells. It has been demonstrated that IDO1 expression is activated after the initiation of maternal blood flow in the intervillous space at the end of the first trimester. It increases through the second trimester and remains elevated at term; however, it is confined to endothelial cells [39]. It is interesting to note that different studies have demonstrated a reduced level of placental IDO expression in PE [40,41,42] despite the increased inflammation which typically characterizes this syndrome and which would be expected to stimulate the kynurenine pathway. This observation further corroborates the critical role played by IDO1 in regulating vascular function within the human placenta.

Recently, a novel kynurenine pathway metabolite was recognized as an endogenous vaso-relaxant factor: the tricyclic L-tryptophan-derived hydroperoxide cis-WOOH. IDO1 forms this factor through the “activation” of oxygen from hydrogen peroxide (H_2_O_2_) to singlet molecular oxygen. High doses of tryptophan were found to reduce the vascular tone in an IDO-dependent mechanism [42]. Therefore, it could be theorized that the downregulation of IDO1 observed in pre-eclamptic patients might contribute to the increased peripheral vascular resistance often present in early PE.

Oxidative stress is well documented as part of the pathology of the placenta [43]. Syncytiotrophoblast stress may be caused by maternal malperfusion secondary to mal-placentation in early pregnancy or secondary to placental growth and compression in late pregnancy [44]. Interestingly, tryptophan metabolism is involved in the generation of the antioxidant response. During the conversion of tryptophan into kynurenine, IDO scavenges reactive oxygen species (ROS), utilizing superoxide anions (O_2_^−^) as a substrate. Contrary to other antioxidants, such as superoxide dismutase, which convert superoxide to another potentially harmful oxidizing compound, such as H_2_O_2_, IDO1 directly removes a pro-oxidant to generate further potent antioxidant compounds. Moreover, various downstream metabolites derived from the catabolism of tryptophan have antioxidant effects: kynurenine, 3-hydroxy-anthranilic acid, 3-hydroxy-kynurenine, anthranilic acid, xanthurenic acid, kynurenic acid, and the phenolic metabolites 3-hydroxy-kynurenine and 3-hydroxy-anthranilic acid [45]. Tryptophan degradation leads to the synthesis of quinolinic acid, which proceeds the generation of nicotinamide adenine dinucleotide (NAD^+^).

When the availability of antioxidant mechanisms is reduced in a low-IDO1 environment, as observed in the PE placentas in this study, the results suggest that cellular homeostasis is severely compromised, and cell stress may occur. In response to stress, the cell may undergo a variety of responses: normality is restored; senescence begins, which means that the cell becomes dysfunctional; programmed cell death occurs; and in the most extreme cases, cell necrosis occurs [44]. Accelerated senescence is known to be present in PE, leading to the activation of mechanisms driving cellular apoptosis [46].

We believe that the broad availability of antioxidant mechanisms is greatly involved with the peculiar architecture of the placenta, a unique organ with two circulations that are in contact with each other. The syncytiotrophoblast covering the chorionic villi, where the fetal blood vessels are running, functions as an endothelium and is exposed to maternal blood. So, it must not only evade aggression from the maternal immune system but also respond to many stresses. The syncytiotrophoblast also acts as a functional barrier, increasing the expression of drug transporters and drug-metabolizing enzymes, as well as nuclear factors and receptors that constitute the most effective mechanism of protection from oxidative-stress-induced injuries.

According to the literature, immunologic maladaptation is deeply involved in the pathogenesis of PE [47,48]. Treg cells, a subpopulation of T cells that suppress the immune response to maintaining self-tolerance, are reduced in PE [47]. HLA-DR, which is involved in graft-versus-host disease, was expressed in the PE placentas but not in the control placentas [48]. It is interesting to note that sperm exposure seems to be important for inducing paternal antigen-specific Treg cells [47]. Indeed, semen increases the amount of immature dendritic cells (DCs) that flow into the uterus from the lymph nodes just after insemination. In contrast, mature DCs flow in the opposite direction to induce the development of Treg cells before implantation. Moreover, cytotoxic T cells are suppressed in a successful pregnancy. On the contrary, in PE, Th1 and Th17 cells are predominant, with a concomitant reduction in Treg cells, thus damaging the immune compromise between the mother and fetus and acting as a root for the development of PE [47]. In this scenario, the AhR acts as a lead player since in a normal pregnancy, its activation promotes immune tolerance by controlling the differentiation of T cells [49]. The activation of the AhR in T cells promotes the expression of the transcription factor Forkhead box P3 (FoxP3), which induces differentiation in Treg cells. By contrast, the activation of the AhR negatively regulates the expression of the transcription factor retinoic acid receptor-related orphan receptor-γt (RORγt), which promotes the differentiation of proinflammatory Th17 cells. However, the starting point may be the activation of the kynurenine pathway in dendritic cells and macrophages, leading to the generation of kynurenine and possibly kynurenic acid, which activate the AhR in an autocrine manner or through the large neutral amino acid transporter (LAT-1) specific to kynurenine on T cells [50].

### 4.3. Clinical Implications

The decreased levels of expression of the AhR, IDO1, the ARNT, TiPARP, and S100A4 in the placental tissues of preeclamptic women relative to controls may be beneficial as potential diagnostic markers for PE. In addition, the altered expression of the AhR pathway suggests that these molecules may be therapeutic targets for PE.

### 4.4. Implications for Research

The presence of the AhR in the nuclei and cytoplasm of the syncytiotrophoblasts indicates that it probably regulates placental function, and the correlations between the AhR, S100A4, and CYP1B1 in normal placentas suggest that these molecules interact in a complex manner. In addition, the presence of the AhR in CD45-positive cells reveals an association between the AhR and immune dysregulation in PE. Additional research is required to validate these findings and develop novel diagnostic and therapeutic strategies for PE.

### 4.5. Strengths and Limitations

These findings may not be directly translated to the in vivo environment because this work focused on ex vivo placental tissue examinations which may not fully reflect the complex in vivo interactions and physiological variables within the placentas of pre-eclamptic women. The study’s cross-sectional approach for placental sampling restricted the investigation of temporal changes or causality. To address this, future research should aim to collect longitudinal data on placental mRNA in maternal blood samples, which will probably reveal how observed mRNA and protein expression variations contribute to the onset and progression of PE. Studies have shown that detecting RNA in the blood of pregnant women can potentially improve the accuracy of PE diagnoses [51]. Furthermore, maternal circulating RNA can help us understand the mechanisms behind PE and offer valuable insights that could lead to new preventive approaches [51]. A study by Tsang et al. (2017) demonstrated that placental mRNA can indeed be collected from maternal blood samples [52]. The researchers identified specific placental mRNA molecules and observed dynamic changes in their expression levels throughout pregnancy. This longitudinal data provided valuable insights into the cellular processes occurring in the placenta. The analysis of placental cellular signature expression in maternal plasma allows for the noninvasive delineation of placental cellular dynamics during pregnancy and the elucidation of extravillous trophoblastic malfunction in early PE [52]. This study should be considered exploratory, and further investigations into the AhR pathway’s role in enzymatic activity and immune system regulation are needed to understand its function in placenta biology. A strength of this study is its ability to identify potential biomarkers for PE by investigating molecular changes in pre-eclamptic women’s placental tissues. The researchers analyzed the AhR using a comprehensive approach, including a qRT-PCR, IHC, and IF, to evaluate gene expression and protein localization. The research also revealed significant links between multiple pathway components and the AhR, offering information about potential interactions and regulatory interconnections in the placental environment. This study’s findings could provide the basis for future research, potentially leading to the development of diagnostic or therapeutic therapies for PE.

## 5. Conclusions

In conclusion, this study sheds light on the molecular alterations associated with PE and suggests potential diagnostic, therapeutic, and mechanistic avenues for enhancing the management of this pregnancy complication. To validate and expand upon these implications, additional research must be conducted.

## Figures and Tables

**Table 1:** This table describes the characteristics of the cases and controls that were analyzed.

Variables	Controls (10)	PE (10)	*p*
Patient characteristics			
Mother’s age (years)	31.00 (29.25–32.75)	33.50 (32.25–36.25)	0.087
Gestational age (weeks)	38.50 (38.00–39.75)	33.50 (31.25–35.75)	<0.05
Nulliparity	50.00% (5/10)	50.00% (5/10)	1.000
Mode of delivery			0.070
Vaginal delivery	80.00% (8/10)	30.00% (3/10)	
Cesarean delivery	20.00% (2/10)	70.00% (7/10)	
Newborn characteristics			
Fetal sex			0.653
Female	50.00% (5/10)	40.00% (4/10)	
Male	50.00% (5/10)	60.00% (6/10)	
SGA 3rd centile	0.00% (0/10)	20.00% (2/10)	0.474
SGA 10th centile	0.00% (0/10)	30.00% (3/10)	0.211
Apgar score, first minute	8.50 (8.00–9.00)	7.00 (7.00–7.75)	0.102
Apgar score, fifth minute	9.00 (9.00–9.00)	9.00 (8.00–9.00)	0.700
Neonatal weight (grams)	3043.00 (2849.00–3341.00)	1746.50 (1166.00–2643.75)	<0.05
Neonatal weight (MoM)	0.93 (0.89–0.98)	0.87 (0.73–0.96)	0.190
Placental weight (grams)	597.50 (502.50–678.25)	265.00 (251.25–505.75)	<0.05
Placental index	0.19 (0.17–0.19)	0.21 (0.18–0.25)	0.315

Acronyms: SGA: small for gestational age; MoM: multiple of the median.

**Table 2:** Here, the qRT-PCR results are displayed as delta-CT expression values.

Variables	Controls (10)	PE (10)	*p*
Expression data			
AhR	1.44 (0.67–2.74)	0.11 (0.04–0.33)	<0.05
CYP1B1	0.03 (0.00–0.35)	0.00 (0.00–0.00)	0.122
IDO1	0.45 (0.16–0.60)	0.02 (0.01–0.08)	<0.05
ARNT	1.19 (0.32–2.40)	0.11 (0.10–0.14)	<0.05
TIPARP	0.27 (0.12–0.65)	0.04 (0.03–0.08)	<0.05
S100A4	0.04 (0.02–0.12)	0.01 (0.00–0.01)	<0.05
AHRR	0.01 (0.00–0.02)	0.00 (0.00–0.01)	0.409

## Data Availability

The data that support the findings of this study are available. However, restrictions apply to the availability of these data, which were used under license for the current study and are not publicly available. Data are, however, available from the authors upon reasonable request and with the permission of the Institutional Review Board.
